# OligoPVP: Phenotype-driven analysis of individual genomic information to prioritize oligogenic disease variants

**DOI:** 10.1038/s41598-018-32876-3

**Published:** 2018-10-02

**Authors:** Imane Boudellioua, Maxat Kulmanov, Paul N. Schofield, Georgios V. Gkoutos, Robert Hoehndorf

**Affiliations:** 1 Computational Bioscience Research Center, King Abdullah University of Science and Technology, Thuwal, Saudi Arabia; 2 Department of Physiology, Development & Neuroscience, University of Cambridge, Cambridge, UK; 3 College of Medical and Dental Sciences, Institute of Cancer and Genomic Sciences, Centre for Computational Biology, University of Birmingham, B15 2TT Birmingham, United Kingdom; 4 Institute of Translational Medicine, University Hospitals Birmingham, NHS Foundation Trust, B15 2TT Birmingham, United Kingdom; 5 NIHR Experimental Cancer Medicine Centre, B15 2TT Birmingham, UK; 6 NIHR Surgical Reconstruction and Microbiology Research Centre, B15 2TT Birmingham, UK; 7 NIHR Biomedical Research Centre, B15 2TT Birmingham, UK

## Abstract

An increasing number of disorders have been identified for which two or more distinct alleles in two or more genes are required to either cause the disease or to significantly modify its onset, severity or phenotype. It is difficult to discover such interactions using existing approaches. The purpose of our work is to develop and evaluate a system that can identify combinations of alleles underlying digenic and oligogenic diseases in individual whole exome or whole genome sequences. Information that links patient phenotypes to databases of gene–phenotype associations observed in clinical or non-human model organism research can provide useful information and improve variant prioritization for genetic diseases. Additional background knowledge about interactions between genes can be utilized to identify sets of variants in different genes in the same individual which may then contribute to the overall disease phenotype. We have developed OligoPVP, an algorithm that can be used to prioritize causative combinations of variants in digenic and oligogenic diseases, using whole exome or whole genome sequences together with patient phenotypes as input. We demonstrate that OligoPVP has significantly improved performance when compared to state of the art pathogenicity detection methods in the case of digenic diseases. Our results show that OligoPVP can efficiently prioritize sets of variants in digenic diseases using a phenotype-driven approach and identify etiologically important variants in whole genomes. OligoPVP naturally extends to oligogenic disease involving interactions between variants in two or more genes. It can be applied to the identification of multiple interacting candidate variants contributing to phenotype, where the action of modifier genes is suspected from pedigree analysis or failure of traditional causative variant identification.

## Introduction

Discrimination of causative genetic variants responsible for disease is a major challenge. An increasingly large family of algorithms and strategies has been developed to aid in identification of such variants^1^. These methods use properties of variants such as evolutionary conservation, predicted structural changes, allele frequency and function to predict pathogenicity. For variants in non-coding sequence regions, additional information used by computational models includes predicted regulatory function and recognized DNA–protein or DNA–RNA interactions^1–3^. Furthermore, phenotype annotations to human and model organism genes can be added to provide another layer of discrimination between involved pathogenic and non-pathogenic variants^4–6^. Phenotype-based methods can identify the likelihood that a particular gene or gene product may give rise to phenotypes observed in an individual^7,8^.

The increasing availability of patient sequence information coupled with resources that provide a detailed phenotypic characterization of diseases, as well as the wealth of gene-to-phenotype associations from non-human disease models^9^, are now enabling new approaches to the prioritization of causative variants and facilitating our ability to dissect the genetic underpinnings of disease^5^. PhenomeNET^10^, developed in 2011, is a computational framework that utilizes pan-phenomic data from human and non-human model organisms to prioritize candidate genes in genetically-based diseases^10^. We have combined PhenomeNET with genome-wide pathogenicity predictions to develop the PhenomeNET Variant Predictor (PVP)^4^ as a system that combines information about pathogenicity of variants with known gene–phenotype associations to predict causative variants. We recently developed the PVP system to classify variants into those likely to be causative or non-causative^4^.

While PVP has a significantly better performance in the prioritization of single variants in monogenic diseases than competing algorithms^4^, the phenotypes of many diseases with a recognized genetic origin show a range of severity, phenotypic spectrum, age of onset and prognosis^11^. While characteristic phenotypic variability can be associated with different alleles in single disease-causing genes or their mode of inheritance, it has been known for some time that in many diseases there is variable expressivity, and in some cases variable penetrance, associated with the same primary mutation in different individuals or pedigrees^12^. This implies that in those cases the phenotypic variability observed must be due to the presence of other modifier variants or environmental influences. Increasingly, several diseases are being understood within the context of complex inheritance and multifactorial disease phenotypes where multiple independent variants modify each other’s effect on phenotype^13^, or, in some cases, render the disease di- or oligogenic where variants in two or more different genes are needed for its clinical manifestation^14^. Evidence for digenic inheritance is available for around 50 diseases^15^ and details are gathered in the Digenic disease database (DIDA)^16^.

Epistatic interactions have been postulated to explain the missing heritability in many types of common and rare disease^12^, and with the increasing clinical use of next generation sequencing, further evidence is accumulating for a spectrum of types of interactions between genes. These interactions are manifest in different ways. For example, there is evidence from population genetics for phenotypic modifier genes such as the modifiers of the age of onset in Huntington’s disease^17,18^, and from a candidate gene approach in Parkinson’s disease^19^. In a similar candidate approach for amyotrophic lateral sclerosis (ALS), affected individuals with proven or potentially pathogenic mutations in two or three known ALS genes are associated again with lower age of disease onset.

Congenital hypothyroidism has both rare monogenic recessive loss-of-function, and common, apparently sporadic, forms. The recent description of patients carrying biallelic and triallelic digenic combinations of variants in known thyroid development and function genes^20^ has lead to the suggestion that a frequent oligogenic origin might explain sporadic hypothyroid cases. Evidence has also been provided for rare trigenic involvement of variants, such as in *TSHR*, *SLC26A4* and *GLIS3*^21^. In addition to these examples of genetic interaction, the observations suggesting digenic/triallelic inheritance in Bardet-Biedel syndrome (BBS)^22^ continue to provoke interest and further research, and illustrate the challenges in establishing formal digenicity^23^. One of the best characterized cases of digenic inheritance is a form of Usher syndrome where digenic heterozygous mutations in *CDH23* and *PCDH15* have been shown to interact in both humans and mice^24^. Other examples are critically discussed elsewhere^15^.

Digenic disease can be divided into two classes: strict digenic disease where variants at both loci are required for the disease, and composite disease which is either the result of the epistatic relationship between alleles of two independent genes modifying the phenotypes of the individual mutations alone, or the phenotypic overlay of two monogenic Mendelian diseases present in the same patient^25,26^. Identification of the genes involved in all of these types of digenic disease usually requires pedigree information or the use of existing knowledge about candidate genes. For example, the selection of candidate genes may rely on the availability of additional information about molecular or functional connections between the entities (genes or gene products) bearing the variants^20^. The difficulties in establishing strong evidence for digenic inheritance are discussed elsewhere^15,27^.

Computational identification of likely causative alleles that are involved in digenic or genetically more complex diseases, in particular for genes not previously associated with the disease, is particularly challenging; such methods would have to be able to incorporate and utilize a large amount of background information about molecular and (patho-)physiological interactions within an organism to determine how combinations of variants jointly result in an observed phenotype. The observation that disease-implicated proteins often interact with each other has stimulated the development of network-based approaches to identification of disease modules^28–31^. However, relevant interactions may occur across much larger distances within pathways and networks, or at the whole organism physiological level where knowledge about biological systems and multi-scale interactions is critical for understanding pathobiology^32,33^. Phenotypes provide a readout for all of these disease-relevant interactions and offer insights into the underlying pathobiological mechanisms^34^. Phenotype data can be a powerful source of information for variant prioritization and is complementary to pathogenicity prediction methods based on molecular information^4,35–38^.

Here, we first evaluate the ability of the PVP system to identify combinations of variants in digenic diseases obtained from a database of digenic diseases. We then present OligoPVP, a novel algorithm for prioritizing digenic or higher order combinations of variants in personal genomes. While the OligoPVP algorithm will not identify whether a phenotype or disease has a digenic or oligogenic inheritance, it identifies and ranks potential causative variants in the same way as PVP but then prioritizes pairs or sets of higher cardinality of potentially interacting variants present in the same genome, as specified by the user, on the basis of prior knowledge about genetic, regulatory and biochemical interactions between them. It is therefore mainly useful to identify candidate causative sets of variants in cases in which digenic or oligogenic inheritance is already suspected, for example due to variable penetrance or expressivity in family studies, or as means of exclusion because established methods for variant prioritization failed.

We apply OligoPVP to the identification of variants in pairs of genes where mutations in two separate genes present in a single individual and lead to a particular phenotypic profile that is not apparent in individuals carrying only one of these variants. We demonstrate that OligoPVP is able to identify gene variant sets in digenic diseases using a set of synthetic whole genome sequences into which we insert multiple known causative gene variants. OligoPVP is freely available at https://github.com/bio-ontology-research-group/phenomenet-vp.

## Materials and Methods

### Digenic disease

The Digenic Disease Database (DIDA) v2^16^ consists of 258 curated digenic combinations representing 54 diseases, with 448 variants in 169 genes. Of the 258 digenic combinations, 189 have Human Phenotype Ontology (HPO) annotations, representing 52 diseases, 153 distinct genes, and 337 unique variants. We use the 189 digenic combinations with HPO annotations in our experiments. 25 of these combinations are triallelic and exhibit compound heterozygosity in one gene while the remaining 164 combinations are biallelic.

We use the combinations of variants from DIDA to generate 189 synthetic whole genome sequences by randomly inserting the causative variants in a randomly selected whole genome sequence from the 1000 Genomes Project^39^.

### Interaction data

We downloaded all interactions occurring in humans from the STRING database version 10.$5$^40^. Then, we mapped all interactions to their respective genes using the mapping file provided by STRING to generate 989,998 interactions between genes, representing 13,770 unique genes. We use these interactions between genes to prioritize combinations of variants in OligoPVP.

### PhenomeNET Variant Predictor

In our work, we use the PhenomeNET Variant Predictor (PVP) version 2.0 (“DeepPVP”)^41^. This version of PVP is based on the Human Phenotype Ontology (HPO)^35^ and the Mammalian Phenotype Ontology (MP)^42^ ontologies obtained from the AberOWL repository^43^ on Feb 7th, 2017. PVP uses gene-to-phenotype associations in humans as well as from the mouse and zebrafish model organisms downloaded on Feb 7th, 2017 from the HPO website^35^, the Mouse Genome Informatics website^42^, and the Zebrafish Information Network website^44^, respectively. The PVP system was trained on data generated from the ClinVar database^45^ accessed on Feb 7th, 2017.

PVP combines features that score the pathogenicity of variants with a phenotype similarity measure that aims to identify whether a variant is likely to cause the phenotypes observed in a patient. Using the cross-species phenotype ontology PhenomeNET^46^, phenotype similarity also can be computed between patient phenotypes and phenotypes in model organisms.

The PVP system used in our analysis, the synthetic genome sequences we generated for the evaluation of our system, and our analysis results can be found at https://github.com/bio-ontology-research-group/phenomenet-vp.

### Evaluation and comparison

We compare PVP and OligoPVP to several state of the art variant prioritization method. Specifically, we compare the PVP and OligoPVP scores to variant pathogenicity prediction scores obtained from CADD v1.3^47^, DANN v1.0^48^, and GWAVA v1.0^49^. Furthermore, we compare our results to the phenotype-based tool Genomiser version 7.2.1^50^ using its default parameters.

## Results

### Prediction of biallelic and triallelic disease variants

We analyze each WGS using the phenotypes provided for the combination of variants in DIDA. We do not filter any variants by minor allele frequency to avoid missing potentially important interacting variants that might have medium to common frequencies in the background population. On average, each WGS in our experiments contains 2,192,967 variants.

We use the phenotypes associated with the combination of variants in DIDA as phenotypes associated with the synthetic WGS, and we use PVP^4^ to prioritize variants, using an “unknown” mode of inheritance model. Out of 164 whole genome sequences where two variants were inserted, we find both causative variants (i.e., the two variants we inserted) as the highest ranked variants in 88 cases (53.7%) and within the top ten ranks in 107 cases (65.2%) (see Table 1). For the 25 cases of triallelic diseases, we find all three causative variants within the first three ranks in 10 cases (40.0%) and we find all three causative variants within the top ten variants in 14 cases (56.0%) (see Table 2). Tables 1 and 2 also compare PVP to established variant prioritization methods, including CADD^47^, DANN^48^, the phenotype-based Exomiser/Genomiser system^50^, and GWAVA^49^. Out of these systems, CADD performs the best in prioritizing combinations of variants; however, PVP can rank variant involved in bi- or triallelic diseases significantly higher than CADD (Mann-Whitney U test, *p* = 6.8 × 10^−5^).

**Table 1:** Comparison of different variant prioritization systems for recovering biallelic variants.

	All			Interacting only		
	Top pair	Top 10 pairs	Combinations	Top pair	Top 10 pairs	Interacting combinations
PVP	88 (53.7%)	107 (65.2%)	164	42 (59.2%)	51 (71.8%)	71
CADD	34 (20.7%)	87 (53.1%)	164	10 (14.1%)	37 (52.1%)	71
DANN	5 (3.1%)	59 (36.0%)	164	0	17 (23.9%)	71
Genomiser	0	0	164	0	0	71
GWAVA	0	0	164	0	0	71
OligoPVP	47 (28.7%)	59 (36.0%)	164	47 (66.2%)	59 (83.1%)	71

We split the evaluation in two parts, one in which we consider all variants and another in which we only consider variants for which we have background knowledge about their interactions.

**Table 2:** Comparison of different variant prioritization systems for recovering triallelic variants.

	All			Interacting only		
	Top triple	Top 10 triple	Combinations	Top triple	Top 10 triple	Interacting combinations
PVP	10 (40.0%)	14 (56.0%)	25	7 (43.8%)	10 (40.0%)	16
CADD	4 (16.0%)	9 (36.0%)	25	7 (43.8%)	12 (75.0%)	16
DANN	0	6 (24.0%)	25	0	4 (25.0%)	16
Genomiser	0	0	25	0	0	16
GWAVA	0	0	25	0	0	16
OligoPVP	10 (40.0%)	10 (40.0%)	25	10 (62.5%)	10 (62.5%)	16

We split the evaluation in two parts, one in which we consider all variants and another in which we only consider variants for which we have background knowledge about their interactions.

Individually, the performance of our approach differs between diseases, depending on the availability of gene–phenotype associations and high quality and informative disease–phenotype associations in DIDA. Table 3 provides an overview of the performance of PVP for individual diseases, and we provide the full analysis results on our website.

**Table 3:** Analysis of top ranks of variants by PVP summarized by disease.

	Top hit	Top 3 hits	Top 10 hits	Variants (Combinations)
Familial long QT syndrome	21 (50.0%)	38 (90.5%)	41 (97.6%)	42 (21)
Kallmann syndrome	18 (47.4%)	27 (71.1%)	27 (71.1%)	38 (19)
Bardet-Biedl syndrome	14 (36.8%)	28 (73.7%)	32 (84.2%)	38 (14)
Alport syndrome	14 (45.2%)	28 (90.3%)	29 (93.6%)	31 (15)
Non-syndromic genetic deafness	12 (50.0%)	18 (75.0%)	18 (75.0%)	24 (12)
Oculocutaneous albinism	6 (40.0%)	12 (80.0%)	12 (80.0%)	15 (7)
Primary ovarian insufficiency	2 (13.3%)	2 (13.3%)	2 (13.3%)	15 (7)
Usher syndrome	5 (33.3%)	11 (73.3%)	12 (80.0%)	15 (7)
Hypodontia	6 (50.0%)	12 (100.0%)	12 (100.0%)	12 (6)
Others	66 (38.2%)	118 (68.2%)	128 (74.0%)	173 (81)

In particular, for the case of hypodontia, PVP identifies all the causative variant pairs in the top 3 ranks in all synthetic patients, and in Familial long QT syndrome, the causitive variant pairs can be found in the top 3 ranks in over 90% of the synthetic patients. Similarly, for the case of Bardet-Biedl syndrome (BBS), PVP ranks 84.21% of causative variant pairs in the top 10, and identifies digenic causative variants in 9 of the 16–20 genes now implicated in BBS^23,51^.

To ensure that newer versions of ontologies and our training data do not already contain, implicitly, information about associations between variants and disease, we perform a semi-prospective experiment; the PVP system we used is based on ontology versions (HPO and MP) and training data obtained on Feb 7th, 2017. We separately test the performance of our system on the digenic cases added to the DIDA database after Feb 7th, 2017. In total, 45 digenic combinations with HPO annotations are newly added to DIDA after the date our PVP system was built. Among these newly added combinations, 42 are biallelic and 3 are triallelic. Table 4 shows the performance of PVP on these cases. We find that the performance on predicting the new cases drops somewhat in comparison to cases before the PVP build date.

**Table 4:** Performance of PVP on all combinations present in DIDA database (All DIDA), combinations added before the PVP build date date of Feb 7th, 2017 (Old DIDA), and combinations added after the cutoff date of Feb 7th, 2017 (New DIDA).

	Biallelic			Triallelic		
	Top pair	Top 10 pairs	Combinations	Top triple	Top 10 triples	Interacting combinations
All DIDA	88 (53.7%)	107 (65.2%)	164	10 (40.0%)	14 (56.0%)	25
Old DIDA	69 (56.6%)	84 (86.9%)	122	10 (45.5%)	14 (63.6%)	22
New DIDA	19 (45.2%)	23 (54.8%)	42	0	0	3

### OligoPVP: Use of background knowledge to find causative combinations of variants

Our results demonstrate that PVP can identify combinations of variants implicated in a disease, outperforming current state-of-the-art gene prioritization approaches. The variants found by PVP are commonly in genes that form a disease module, i.e., a set of interacting genes that are jointly associated with a disease or phenotype^52^. For example, out of the 164 biallelic combinations used in our study, we can find evidence of interactions for 71 biallelic combinations and 16 triallelic combinations using the interaction database STRING^40^. The STRING resource contains background knowledge about the interaction between genes based on protein-protein interactions, co-expression, pathway involvement, or co-mention in literature, and therefore provides a wide range of distinct interaction types which may underlie a phenotype. We have now exploited this background knowledge to further improve prioritization of variants in oligogenic diseases which involve interactions between alleles of two or more genes.

OligoPVP is an algorithm that uses background knowledge from interaction networks to prioritize variants in oligogenic diseases. It identifies likely causative variants in interacting genes and ranks tuples of *n* variants in genes that are connected through an interaction network. OligoPVP will first rank all variants in a set of variants (such as those found in a VCF file) independently using PVP and assign each variant *ν* a prediction score *σ*(*ν*). When ranking combinations of *n* variants, OligoPVP will then evaluate all *n*-tuples of variants ${v}_{1},\,\mathrm{...,}\,{v}_{n}$ and assign a score $\bar{\sigma }$ to the *n*-tuple $({v}_{1},\,\mathrm{...,}\,{v}_{n})$, given an interaction network ϒ:

$$\bar{\sigma }({v}_{1},\,\mathrm{...,}\,{v}_{n})=\{\begin{array}{ll}\sigma ({v}_{1})+\mathrm{...}+\sigma ({v}_{n}) & {\rm{if}}\,{v}_{1},\,\mathrm{...,}\,{v}_{n}\,{\rm{are}}\,{\rm{variants}}\,{\rm{in}}\,{\rm{a}}\,{\rm{connected}}\,{\rm{subgraph}}\,{\rm{of}}\,\Upsilon \,\\ 0 & otherwise\end{array}$$

Algorithm 1 illustrates the procedure to find oligogenic disease modules in more detail. OligoPVP can identify combinations of variants both in exonic and non-exonic regions. For non-exonic variants, we assign the gene that is located closest to the variant as the variant’s gene.

**Algorithm 1:**
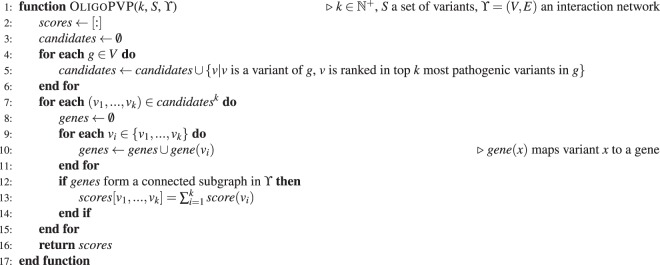
OligoPVP prioritization of oligogenic combinations.

The OligoPVP algorithm strictly relies on an interaction network as background knowledge and will not prioritize any combinations of variants if they are not connected in a known network. For digenic or oligogenic disease we assume that the interactions between alleles are mediated directly or indirectly through physical or regulatory mechanisms at any level, e.g., through DNA (for example transcriptional regulation), RNA (for example processing of RNA or half life modulation), or protein (e.g., direct functional interaction or co-participation in the same pathway or physiological processes). Data relevant to all these levels of interaction is available in STRING. OligoPVP is of course limited by the completeness of the interaction data available and until more complete high level physiological modeling is achieved, interactions at the level of the whole organism physiome will be difficult to capture.

OligoPVP utilizes beam search^53^ to optimize memory usage. We can simply extend OligoPVP to also consider compound heterozygote combinations of variants by adding self-loops to each node in ϒ. The main advantage of OligoPVP is its ability to identify and rank connected sets of variants higher than individual variants. Table 5 lists several cases in which OligoPVP prioritizes pairs of variants higher than PVP would prioritize them on their own. On the other hand, OligoPVP will not prioritize combinations of variants if they are in genes that are not connected in the background network ϒ. Supplementary Table 1 lists some of the cases which can be prioritized with PVP but not OligoPVP.

**Table 5:** Cases of DIDA combinations improved by OligoPVP in comparison to PVP. OligoPVP incorporates protein-protein interactions in the prioritization of variant tuples.

DIDA ID	Gene A	Gene B	Disease name (ORPHANET)	PVP Rank A	PVP Rank B	OligoPVP Rank
dd225	PSMA3 (c.696_698delAAG)	PSMB8 (c.224C > T)	CANDLE syndrome	8	1	1
dd226	PSMA3 (c.404 + 2T > G)	PSMB8 (c.224C > T)	CANDLE syndrome	292	1	2
dd228	PSMB4 (c.666C > A)	PSMB8 (c.313A > C)	CANDLE syndrome	1980	1	2
dd159	EMD (c.110_112delAGA)	LMNA (c.892C > T)	Familial atrial fibrillation	1	21	4
dd043	SCN1A (c.5054C > T)	SCN2A (c.1571G > A)	Generalized epilepsy with febrile seizures-plus	1	7	2
dd114	CD2AP (c.1488G > A)	NPHS2 (c.622G > A)	Familial idiopathic steroid-resistant nephrotic syndrome	1	141	4
dd053	KCNE1 (c.379C > A)	KCNQ1 (c.1022C > T)	Familial long QT syndrome	30	1	4
dd229	CDK5RAP2 (c.4187T > C)	CEP152 (c.3014_3015delAAinsT)	Seckel syndrome	22	1	5
dd007	PCDH15 (c.5601_5603delAAC)	CDH23 (c.193delC)	Usher syndrome	7	1	1
dd052	HAMP (c.212G > A)	HFE (c.845G > A)	Rare hereditary hemochromatosis	22	1	3

We compare the results of applying OligoPVP to the ranks obtained using PVP on individual variants.

## Discussion

With the increasing appreciation of the relationship between complex and Mendelian diseases^54^, the ability to discover multiple variants contributing to disease phenotype in the same genome provides a powerful tool to help understand the genetic architecture of diseases and the underlying physiological pathways. With the advent of whole exome and whole genome sequencing, advances have been made using existing approaches to prioritize causative variants. However, use of standard criteria for the identification of rare disease variants, e.g., a low minor allele frequency (MAF) of, for example, less than 1%, are designed to detect *de novo*, homozygous, or compound heterozygous variants, and may not give sufficient priority to variants of low apparent pathogenicity, haploinsufficiency, or low to medium MAF, although these variants may still be important in the pathogenesis of a disease. Because the approach we take with OligoPVP and PVP makes no assumptions about allele frequency or mode of inheritance, and balances estimates of pathogenicity with phenotypic relatedness, a wider net is cast and candidate genes affecting the penetrance, expressivity or spectrum of the phenotype are more readily identified.

Genes whose variants contribute to a disease phenotype are considered likely to be situated within the same pathway or network^55–58^. In addition to well established studies of genes involved in, for example the ciliopathies^23,59^, newer studies are now identifying network relations between genes implicated in the oligogenic origins of diseases^60,61^. Consequently, we can exploit background knowledge on the interactions of gene products in OligoPVP and improve the ranking of candidate pairs of variants over that assigned through pathogenicity and phenotypic relatedness scores alone.

Currently, identification of multiple variants contributing to the characteristics of a disease in a cohort or individual patient rely either on a candidate gene approach or the assumption that contributing alleles are likely to be rare in the population. The contribution of rare alleles of low effect, i.e., which by themselves generate sub-clinical phenotypes, for example hypomorphs, may be missed in this way, and rare to medium frequency alleles which modify the penetrance or expressivity of a second remain difficult to identify (the former because of low potential pathogenicity and the latter because of high frequency and lack of association with a phenotype when occurring alone). An alternative strategy for identification of candidate genes for highly heterogeneous human diseases is to use mouse genetics to identify phenotypic modifier genes. For example, neural tube defects are believed to involve more than 300 genes in the mouse, mutations in many of which need to be digenic or trigenic for expression of the phenotype^62^. Similarly, mouse double heterozygous mutants in *Nkx2-1* and *Pax8* show strain-specific thyroid dysgenesis phenotypes not seen in the individual mutations^63^. The scale of genetic interactions becoming apparent from mouse studies strongly supports the suggestion that in the human, we are only seeing the tip of a very important iceberg^64^.

The OligoPVP algorithm offers a generic framework for using background knowledge about any form of interaction between genes and gene products to guide the identification of combinations of variants. In its generic form, the worst case complexity of the algorithm is ${\mathscr{O}}({n}^{k})$ where *n* is the number of variants and *k* the size of the module (the size of the module is a parameter of OligoPVP). It is clear that our algorithm, in its basic form, will not yet scale to large disease modules (i.e., large *k*); however, in the future, several methods can be used to further improve the average case complexity to find larger disease modules.

Furthermore, background knowledge about interactions between genes and gene products is far from complete. In particular, information about coarse scale physiological interactions, i.e., those that occur based on whole organism physiology, are significantly underrepresented in interaction databases^32^. Additionally, interaction networks may have biases such as over-representation of commonly studied genes^65,66^, and these biases will likely effect the performance of our algorithm. As more genomic data related to complex diseases becomes available, more work will be required to identify and remove these biases in the identification of phenotype modules from personal genomic data.

The function of OligoPVP is not to determine whether a disease is formally di- or oligogenic but to assess whether sets of variants may be jointly responsible for phenotype when an individual contains multiple, potentially pathogenic, variants in two or more genes that might, from background knowledge, be expected to interact to generate the phenotypic profile of the patient. The user is able to specify the cardinality of the set of variants that should be prioritized, and the rank scores provide a relative measure of the likelihood that two, three or more specific allelic variants might be involved.

While this limitation restricts OligoPVP’s applicability, we nevertheless believe our algorithm to have important applications. The most likely scenario in which OligoPVP can be used successfully is to generate hypotheses for combinations of specific variants after other variant prioritization approaches have failed to yield significant results. Alternatively, knowledge of family history or ethnic background might suggest the contribution of two or more genes to the disease phenotype, and OligoPVP can then be used to identify plausible combinations of genes and allelic variants.

OligoPVP is, to the best of our knowledge, the first phenotype-based method to identify disease modules in personal genomic data. With the large (i.e., exponential) number of combinations of variants that have to be evaluated in finding disease modules, it is clear that any computational method has to make use of background knowledge to restrict the search space of potentially causative combinations of variants. OligoPVP is such a method which uses knowledge about interactions and phenotype associations to limit the search space. In the future, more background knowledge can be incorporated to improve OligoPVP’s coverage as well as accuracy. OligoPVP is freely available at https://github.com/bio-ontology-research-group/phenomenet-vp.

## Electronic supplementary material

Supplementary Table 1
