# Supplementary material for: OligoPVP: Phenotype-driven analysis of individual genomic information to prioritize oligogenic disease variants
Source: Sci Rep. 2018 Oct 2;8:14681. doi: 10.1038/s41598-018-32876-3 (PMC6168481; doi:10.1038/s41598-018-32876-3)
Supplement: Supplementary file 1 — Supplementary Table 1 [file 41598_2018_32876_MOESM1_ESM.pdf]

OligoPVP: Phenotype-driven analysis of individual genomic information to prioritize oligogenic disease variants

Imane Boudellioua[1], Maxat Kulmanov[1], Paul N Schofield[2], Georgios V Gkoutos[3,4,5,6,7], and Robert Hoehndorf[1,*]

[1]Computational Bioscience Research Center, King Abdullah University of Science and Technology, Thuwal, Saudi Arabia
[2]Department of Physiology, Development & Neuroscience, University of Cambridge, Cambridge, UK
[3]College of Medical and Dental Sciences, Institute of Cancer and Genomic Sciences, Centre for Computational Biology, University of Birmingham, B15 2TT, Birmingham, United Kingdom
[4]Institute of Translational Medicine, University Hospitals Birmingham, NHS Foundation Trust, B15 2TT, Birmingham, United Kingdom
[5]NIHR Experimental Cancer Medicine Centre, B15 2TT, Birmingham, UK
[6]NIHR Surgical Reconstruction and Microbiology Research Centre, B15 2TT, Birmingham, UK
[7]NIHR Biomedical Research Centre, B15 2TT, Birmingham, UK
[*]robert.hoehndorf@kaust.edu.sa, +966-54-0523450

Table 1. Cases of DIDA combinations missed by OligoPVP prioritization incorporating protein-protein interaction network data, in comparison to ranks of individual variants of each digenic combination ranked by DeepPVP.

DIDA ID	Gene A	Gene B	Disease name (ORPHANET)	DeepPVP Rank A	DeepPVP Rank B	OligoPVP Rank
dd010	NEK1 (c.1640insA)	DYNC2H1 (c.11747G>A)	Short rib-polydactyly syndrome	1	2	Not Found
dd211	MYH7 (c.2645A>G)	RBM20 (c.2062C>T)	Familial isolated dilated cardiomyopathy	2	1	Not Found
dd040	GDAP1 (c.358C>T)	MFN2 (c.479_480delTG)	Charcot-Marie-Tooth disease	1	2	Not Found
dd125	EDA (c.769G>C)	WNT10A (c.511C>T)	Hypodontia	1	2	Not Found
dd218	BMPR2 (c.1471C>T)	KCNA5 (c.1448del)	Heritable pulmonary arterial hypertension	1	2	Not Found
dd244	NLRP3 (c.526C>T])	MEFV (c.442G>C)	Familial Mediterranean fever	2	1	Not Found
dd028	MITF (c.824delA)	TYR (c.1205G>A)	Ocular albinism with congenital sensorineural deafness	1	2	Not Found
dd013	NSMF (c.1132-22_1132--15del)	FGFR1 (c.1025T>C)	Kallmann syndrome	2	1	Not Found
dd018	FGFR1 (c.165_171del)	PROKR2 (c.518T>G)	Kallmann syndrome	1	2	Not Found
dd164	FGFR1 (c.1042G>A)	IL17RD (c.1136A>G)	Kallmann syndrome	1	2	Not Found
dd165	KISS1R (c.581C>A)	IL17RD (c.2204C>T)	Kallmann syndrome	1	2	Not Found
dd166	FGFR1 (c.2075A>G)	DUSP6 (c.545C>T)	Kallmann syndrome	1	2	Not Found
dd168	SPRY4 (c.722C>A)	DUSP6 (c.1037C>T)	Kallmann syndrome	1	2	Not Found
dd169	SPRY4 (c.722C>A)	FGFR1 (c.1447C>A)	Kallmann syndrome	2	1	Not Found
dd117	TYR (c.230G>A)	SLC45A2 (c.1045G>A)	Oculocutaneous albinism	1	2	Not Found
dd121	TYR (c.346C>T)	OCA2 (c.1441G>A)	Oculocutaneous albinism	1	2	Not Found
dd123	LMBRD1 (c.1056delG)	MTR (c.3518C>T)	Homocystinuria without methylmalonic aciduria	2	1	Not Found
dd116	WT1 (c.1228+5G>A)	NPHS1 (c.1126C>G)	Familial idiopathic steroid-resistant nephrotic syndrome	1	2	Not Found
dd050	HMBS (c.422+1G>T)	UROD (c.650_651dupTT)	Porphyria	2	1	Not Found
dd001	KCNQ1 (c.1022C>A)	KCNH2 (c.2592+1G>A)	Familial long QT syndrome	1	2	Not Found
dd048	KCNE2 (c.178T>C)	SCN5A (c.4868G>A)	Familial long QT syndrome	2	1	Not Found
dd065	KCNE1 (c.95G>A)	SCN5A (c.4931G>A)	Familial long QT syndrome	2	1	Not Found
dd069	SCN5A (c.5455G>A)	KCNH2 (c.298C>G)	Familial long QT syndrome	1	2	Not Found
dd134	ATP2B2 (c.1756G>A)	MYO6 (c.737A>G)	Non-syndromic genetic deafness	1	2	Not Found
dd145	KCNJ10 (c.1042C>T)	SLC26A4 (c.919-2A>G)	Non-syndromic genetic deafness	2	1	Not Found
dd206	GJB2 (c.35delG)	TMPRSS3 (c.208delC)	Non-syndromic genetic deafness	2	1	Not Found
dd124	MYO7A (c.2311G>T)	PCDH15 (c.158-1G>A)	Usher syndrome	2	1	Not Found
dd262	TEK (c.309A>C)	CYP1B1 (c.343G>C)	Congenital glaucoma	2	1	Not Found
dd026	MYOC (c.1196G>T)	CYP1B1 (c.1103G>A)	Juvenile glaucoma	2	1	Not Found
dd171	ITGA7 (c.2656G>A)	MYH7B (c.2668C>T)	Left ventricular non-compaction	2	1	Not Found
dd224	TRIM54 (c.316G>A)	TRIM63 (c.739C>T)	Congenital myopathy with protein accumulation	1	2	Not Found
dd019	BAAT (c.226A>G)	TJP2 (c.143T>C)	Familial hypercholanemia	1	2	Not Found
dd006	ATP2B2 (c.1756G>A)	CDH23 (c.5663T>C)	Non-syndromic genetic deafness	1	2	Not Found
